# Developing and implementing mental health policy in Zanzibar, a low income country off the coast of East Africa

**DOI:** 10.1186/1752-4458-5-6

**Published:** 2011-02-14

**Authors:** Rachel Jenkins, Mahmoud Mussa, Suleiman A Haji, Mohammed S Haji, Ahmed Salim, Said Suleiman, Alya S Riyami, Abdul Wakil, Joseph Mbatia

**Affiliations:** 1 Department of Health Services and Population Research, King's College London, Institute of Psychiatry, David Goldberg Centre, De Crespigny Park, London, UK; 2 Department of Community Mental Health Services, Ministry of Health and Social Welfare, Zanzibar, Tanzania; 3 Saidia Wagonjwe Wa Akili Zanzibar (SWAZA), Zanzibar, Tanzania

## Abstract

**Background:**

The Zanzibar Ministry of Health and Social Welfare, concerned about mental health in the country, requested technical assistance from WHO in 1997.

**Aims:**

This article describes the facilitation over many years by a WHO Collaborating Centre, of sustainable mental health developments in Zanzibar, one of the poorest countries in the world, using systematic approaches to policy design and implementation.

**Methods:**

Based on intensive prior situation appraisal and consultation, a multi-faceted set of interventions combining situation appraisal to inform planning; sustained policy dialogue at Union and state levels; development of policy and legislation, development of strategic action plans, establishment of intersectoral national mental health implementation committee, establishment of national mental health coordination system, integration of mental health into primary care, strengthening of primary-secondary care liaison, rationalisation and strengthening of secondary care system, ensuring adequate supply of medicines, use of good practice guidelines and health information systems, development of services for people with intellectual disability, establishment of formal mechanism for close liaison between the mental health services and other governmental, non-governmental and traditional sectors, mental health promotion, suicide prevention, and research and development.

**Results:**

The policy and legislation introduced in 1999 have resulted in enhanced mental health activities over the ensuing decade, within a setting of extreme low resource. However, advances ebb and flow and continued efforts are required to maintain progress and continue mental health developments. Lessons learnt have informed the development of mental health policies in neighbouring countries.

**Conclusions:**

A multi-faceted and comprehensive programme can be effective in achieving considerable strengthening of mental health programmes and services even in extremely low resource settings, but requires sustained input and advocacy if gains are to be maintained and enhanced.

## Background

While other regions of the world are making economic progress with accompanying improvements in health indices, poverty and low life expectancy remain major problems in sub-Saharan Africa (SSA) with a doubling of the number of people living in poverty in Africa in the last 20 years [1]. Zanzibar, comprising two main islands, Unguja (normally known as Zanzibar island) and Pemba, with a constellation of surrounding small islets, has a population of 1.2 million. It is one of the poorest countries in SSA with malnutrition, political conflict, and growing problems of HIV and substance abuse. Zanzibar's original settlers were Bantu speaking Africans from the mainland. Persians arrived from the tenth century onwards, and in later centuries Arabs arrived, especially from Oman, to trade in ivory, slaves and spices.

Zanzibar provided a base for the Omani Arabs to control 1,000 miles of the mainland coast from present day Mozambique to Somalia. Indeed, in 1832, Sultan Seyyid Said moved his Sultanate from Muscat to Zanzibar which remained part of Oman until Zanzibar became independent in 1963 and, since 1964, it became part of the Union of Tanzania Mainland and Zanzibar. Widespread intermarriage between Shirazi Persians and Africans gave rise to a coastal community with distinctive features and a language derived in part from Arabic, which became known as Swahili (derived from the Arab word Sawahil meaning coast). The Zanzibar descendants of this group were mainly involved in agriculture and fishing. Those Shirazi who did not intermarry retained their identity as a separate group. Indian traders arrived for the spice and ivory trade, and settled as shop keepers, traders, artisans and professionals. The British became involved in missionary and trading activities in east Africa and attempted to suppress the slave trade centered in Zanzibar. With these influences, Zanzibar has become predominantly Islamic (97%) - the remaining 3% is made up of Christians, Hindus and Sikhs.

Due to its historical roots, Zanzibar retains its own President, Revolutionary Government, House of Representatives, and some ministries, including the Ministry of Health and Social Welfare (MOHSW).

Mental health is not often perceived by governments as a priority issue in resource poor settings, but in 1997 the deputy health minister for Zanzibar requested expert assistance from the WHO "Nations for Mental Health" [2,3], for the preparation and implementation of mental health policy and legislation for Zanzibar. Technical support was given by the first author RJ, Director of the WHO Collaborating Centre at the Institute of Psychiatry, London (WHOCC) at the request of WHO HQ, in collaboration with WHO AFRO, and was followed by continued support by RJ/WHOCC over the ensuing decade, in close liaison with the Zanzibar and Tanzania mainland respective Ministries of Health and Social Welfare and WHO country offices. This paper describes an integrated approach to mental health policy development and implementation in Zanzibar 1999-2009, combining detailed situation appraisal, integrated mental health policy and plans; mechanisms for sustainable implementation, using locally available resources and integrated into local systems; and monitoring to fine tune implementation (see additional file 1).

## Methods

A multi-faceted and comprehensive programme was instituted which combined situation appraisal to inform planning, sustained policy dialogue at national level, strengthening a mental health service coordination system, supervision and training, development workshops, production of toolkits, development of guidelines, and establishment of intersectoral partnerships. Local collaborators and key stakeholders were involved at every stage, with whom information from the appraisal was regularly discussed, enabling regular triangulation of findings, and contributed to fine tuning of the strategic action plans.

Situation appraisal included analysis of available data and documents on general health and mental health issues in Zanzibar, rapid situational assessment, using an approach drawing on previous work in infectious diseases [4-12], and which has subsequently underpinned the development of similar work in mental health elsewhere, and included site visits, discussions with key stakeholders and key informant interviews covering contextual issues (social, geographic, political, historical, cultural), needs, service structures, resources, processes and outcomes; as well as further examination of documents, and routine data. Key informant interviews, focus groups and direct observation of clinical practice were used to explore attitudes towards mental illness; contextual, and health system barriers to change and care delivery, especially those factors which hindered inter-sectoral approaches and the engagement of users and civil society organisations in the planning and delivery of care. Once analysed, the information from this rapid appraisal informed the third stage of sustained policy dialogue, resulting in the development of a multifaceted set of interventions, tailored to the country context, and formulated into policy, legislation and strategic action planning (see additional file 2). The strategy included the establishment of a national intersectoral mental health implementation committee, establishment of a national mental health coordination system (see Figure 1 in additional file 3), integration of mental health into primary care, strengthening of primary-secondary care liaison, rationalisation and strengthening of secondary care system, ensuring adequate supply of medicines, use of good practice guidelines and health information systems, development of services for people with intellectual disability, establishment of formal mechanism for close liaison between the mental health services and other governmental, non-governmental and traditional sectors, mental health promotion, suicide prevention, and research and development. The fourth stage, which has so far lasted ten years, has comprised sustained implementation, regular review of progress and constraints, fine tuning action plans, and continued dialogue with stakeholders.

## Results

### Situation appraisal

Many if not most people with mental disorders consult **t**raditional health practitioners (THPs), who are common across Zanzibar [13-15], have a professional association, "u Jumuiya ya Watoaji Huduma za Matibabu ya Asilia Zanzibar" and are registered with the MOHSW. Discussions with traditional healers on both Unguja and Pemba indicated that they were familiar with psychosis, different forms of epilepsy and alcohol abuse; that they had clear views of what they could manage and what was best dealt with by the hospital, and that they would often refer clients to the general or mental hospital for hospital tests and treatment.

### Primary care

In 1997, Zanzibar already had a functioning primary health care system staffed by nurses and health educators, with regular continuing professional development (CPD), but the primary care system was focused almost exclusively on physical health, and its information collection form contained 34 categories for physical disorders and only one overall category for mental disorders, which exemplified and encouraged neglect of mental disorders in planning. Links between primary and secondary care were not systematic, and primary care had no medicines for mental disorders or epilepsy. However, all male nurses graduating from the College of Health Sciences had done one year's psychiatric training as part of their 4 year basic training, and so most PHCUs contained staff with some mental health expertise.

### Secondary care

The secondary care system for physical health consisted of a national hospital Mnazi Moja on Unguja and three district hospitals in the towns of Chake Chake, Wete, and Mkoani on Pemba). The secondary care system for mental health consisted of an inpatient hospital, Kidongo Chekundu, (KC) for Unguja but no inpatient unit for Pemba; and outpatient departments (OPD) on both Unguja and Pemba. The structural environment for mental health inpatient care was highly disadvantaged relative to physical inpatient care, with holes in the roof letting in rain, blocked drains, unhygienic toilets, and no water supply. Half the beds had no mattresses, sheets, blankets, or pillows. There were no mosquito nets, and bed bugs and cockroaches were prevalent. The in-patients had no regular activity programme and Occupational Therapy (OT) was only available for around 11 out of the 120 inpatients. There were no available psychological and social treatments. The hospital diet was inadequate in quantity and quality (consisting of only rice and beans), relying on supplementation by relatives. Many patients had obvious malnutrition and vitamin deficiencies, including scurvy. Hospital cooking facilities were unhygienic and dangerous, and there was frequently no money available for cooking fuel.

The mental hospital had no means of communication, and no transport for transferring patients from the port (for referrals from Pemba) or to the general hospital (for severe physical illness). Security at the hospital was a problem, with theft from the site and sexual harassment of women. The supply of essential psychotropic medication was inadequate, resulting in patients remaining untreated and hence unable to return home. There was also difficulty in obtaining antibiotics and other physical treatment for psychiatric patients, especially those with TB whom the general hospital refused to treat or to send the proper supply of medicines. Co-ordination between primary and secondary care was haphazard, and there were no good practice guidelines for mental disorders. The KC annual report to the MOHSW gave no account of the high mortality in the inpatient unit, and indeed hospital staff were unaware of the total as there was no collation and review of the data. There were no case registers of clients with complex needs who required sustained follow up. All patient admissions were theoretically on a section; the Act (Cap 72 section 5) did not allow for voluntary admissions.

The human resource situation in the specialist service was inadequate, with too few nurses, no permanent fully qualified occupational therapist, social worker or psychologist. The only psychiatrist in Zanzibar was semi-retired, but assisted the KC outpatient clinic one day a week. All inpatients were assessed and treated by nurses. There were no psychiatrists or trained social workers available to give independent opinions on involuntary admission, and no review tribunals for patients to appeal against involuntary admission. Stakeholders identified a pressing need for access to marital therapy and adequate treatment because under Islamic religious law, a person may readily obtain a divorce if the spouse has a mental illness, resulting in major social and economic vulnerability, especially of female clients.

### Human resource development

Since 1985, the College of Health Sciences has produced "nurse psychiatrists" and nurse midwives (each with 3 years general training and 1 year specialist training), and this course has recently been reduced to three years duration in total, with all students studying both mental health and midwifery. In addition, 4 batches of community health nurses were produced 1994-1998 with only 1 year training. The graduates of the College of Health Sciences are used to staff primary and secondary care posts across both Unguja and Pemba, and are expected to prescribe in both primary and secondary care, to cope with the lack of fully qualified doctors (none in primary care and few in secondary care). The MOHSW obtained the overseas training of one psychiatrist in the 1970s, who returned to Zanzibar, leading the service through the 1980s and early 1990s, but took early retirement and so by 1998, it had none (except 1 voluntary day a week from the retired psychiatrist), nor any in training. Zanzibar also had no sustainable plan to develop or recruit occupational therapists, psychologists and social workers. The training of one social worker was arranged who immediately went to work elsewhere, and one nurse had received a one year occupational therapy course on the mainland. Stakeholders reported that staff consider it as a punishment to be sent to work in the mental hospital, and that the MOHSW sometimes uses it as a place to employ ineffective workers, making it difficult to recruit quality nursing staff and effective managers.

### Child services and intellectual disability

The Ministry of Education has a division of special education and started a special education programme in 1998, with Unguja and Pemba each having a special education unit containing 2 specialist teachers, aiming to rehabilitate children with intellectual disability back to normal schools. The special education units teach for 4 hours a day, organise field trips and do special Olympic games. Many of the children have specific neurological problems such as cerebral palsy and epilepsy, but the supply of medicines for epilepsy is inadequate. Many also have specific psychological problems, some of which are referred to KCH outpatient clinic but there is no specific dedicated provision in the current psychiatric services for either normal or intellectually handicapped children, and there is no speech therapist in Zanzibar.

### Police and prisons

There is liaison with the prisons which aim to transfer most prisoners with psychosis to KC. Police are often called upon to transfer psychotic patients in the community to hospital and to provide cell accommodation while awaiting funds from the MOHSW to pay for the ferry from Pemba to Unguja. There were some allegations of mistreatment, and no clarity on whose responsibility it is to provide food for people with mental illness in a police cell, so that often the responsible nurse had to find the food out of his own pocket.

### The general health education programme

By 1997, the general health education programme was already establishing good links to schools, the media and health workers. There was an addiction programme which already had links to the school health programme, youth to the Ministry of Education, women and children youth and tourism, and labour Ministries.

A mental health NGO, Saida Wagonjwa wa Akil Zanzibar (Help Mentally Ill People in Zanzibar, SWAZA) had recently been started, and by 1998 was fundraising and collaborating with the MOHSW and other NGOs.

### Morbidity and Mortality

An epidemiological household survey of schizophrenia and epilepsy was conducted in the 1980s [15]. There was no official mortality data collection in Zanzibar and senior Ministry of Health officials indicated that rates of suicide were extremely low. However, suicide is a taboo in Zanzibar, suicide is believed to prevent entry to heaven, and it remains a crime (Cap 7, Section 5 of 1948), resulting in general under reporting of suicide, lack of proper care and attention to deliberate self harm, lack of education about suicide risk management, and reduced access to help for patients and families. Stakeholder consultations revealed a number of young people (especially pregnant unmarried teenagers) who had killed themselves jumping from high buildings, and people with psychosis who had recently killed themselves by jumping off the ferry while on their way to be admitted to KC, underlining the need for community awareness, mental health promotion in schools, suicide risk assessment and management in primary care and specialist staff, and also underlines the need for an inpatient unit on Pemba. Local TV also reported extensive details of suicides, and their methods, a practice likely to aggravate suicide rates [16].

The overall mortality figures at the mental hospital were extremely high, sometimes related to epidemics of cholera [17], and reviews of cholera on Zanzibar [18] had not investigated the high cholera mortality at the mental hospital, another example of exclusion from the health mainstream, with major consequences for client outcomes.

### Governmental attitudes

The situational analysis identified a number of governmental attitudes and beliefs likely to impede the implementation of the project aims, including a narrow understanding of mental health policy as solely hospital provision of mental health services; lack of recognition of the scale of population morbidity, and of the important potential for decentralized interventions outside the hospital; a view that the grossly inadequate conditions within the mental hospital were somehow the fault of the clients themselves (e.g. the inadequate food supply was caused by psychiatric patients eating more than other people); and that mental health was not sufficiently important to warrant solutions to the problems of shortage of essential drugs, no reliable communication or transport, and limited number of qualified personnel. In contrast, the community population and mental health staff recognised the multi-factorial causation and consequences of mental illness, and the potential for community interventions [13].

### Governance and financing of the mental health programme

Mental illness was not well represented in the MOHSW with no full-time representative, no clear lines of accountability, and no national mental health co-ordinator. Such current attention as there was within the MOHSW was focused on the mental hospital, especially its shortage of food, cooking fuel and blocked drains, but not on the wider mental health programme. Thus mental health expenditure, which was subsumed into the budget for Mnazi Moja hospital, was entirely focused on the inpatient unit, and was completely inadequate even to support that. Financing was needed for three parallel streams of activity: community action to promote mental health, primary care of mental disorders, and decentralized specialist services. In 1999, as part of a wider health sector reform project for Zanzibar planned by the African Development Bank (ADB), RJ was commissioned to produce a funding proposal for mental health services in Zanzibar. This was developed in collaboration with MM and other Zanzibar stakeholders.

### Subsequent Policy, Legislation, Coordination and Service Delivery

The issues identified in the 1998 situation appraisal were considered and addressed in the draft policy which was extensively reviewed by stakeholders, revised and then passed by the House of Representatives [19]. The policy set out the need for mental health and substance abuse legislation which were produced and passed [20,21], which were all endorsed by the President of the Zanzibar Revolutionary Government. The substance abuse legislation has since been revised [22].

Organisational changes proposed in the mental health policy included measures to enhance the capability of the Ministry to Implement the Mental Health and Substance Abuse Programme, measures to strengthen primary care and secondary care, and their linkages, linkages with other sectors, and measures to enhance availability of human resource. (Additional file 1 sets out the five major domains for policy implementation and additional file 2 sets out the detailed outputs and activities covered in the mental health policy).

The capability of the Ministry of Health to implement the Mental Health and Substance Abuse Programme was strengthened by the establishment of a mental health coordinating system, a national intersectoral committee to steer implementation, and mutual collaboration with the Mainland. The mental health coordinating system was designed to provide overall leadership and presence in the MOHSW, accountable to the minister; coordination of the mental health programme in both Unguja and Pemba, and detailed implementation of primary care and secondary care of mental disorders, and public mental health promotion and education (see additional file 3). The national mental health coordinator was appointed immediately, and the zonal coordinators followed shortly after. A separate national coordinator was appointed for substance abuse, but with the intention of eventually merging the mental health and substance abuse programmes. The primary care and community coordinators were appointed in 2001-2 but some left the country for additional training, and replacements have been difficult without enhanced salaries for the additional responsibilities, especially in Pemba. The MOHSW obtained two UN volunteer psychiatrists who were posted to KC and Pemba for several years, coordinating specialist services, but UN rules prevent the long term continuation of these psychiatric postings to Zanzibar.

Considerable advocacy has been needed to ensure that the national mental health coordinator is included in relevant generic health sector meetings, and while this is now largely routine, the coordinator is still occasionally excluded from some key committees. The national mental health implementation committee was established, but has never been allocated MOHSW funds, and has only occasionally obtained external financial support. Its role has therefore now been merged with that of the mental health legislation board which has received sporadic MOHSW funding. It has been difficult for Pemba colleagues to attend, or for meetings to be held in Pemba through lack of a travel budget. Collaboration with the Tanzania mainland programme for mental health and substance abuse programme was initiated, funded by the mainland MOHSW, and there have now been a number of joint strategy development and training activities. Although funds for mental health were agreed within the ADB lending programme for Zanzibar, subsequent staff and policy changes within the ADB headquarters resulted in the funds never being allocated to mental health until this year when the ADB plans to build an inpatient unit for Pemba.

Measures to strengthen Primary Mental Health Care included recommendation of inclusion of Mental Health into the Continuing Education Programs, development of good practice guidelines, an adequate supply of essential medicines, the inclusion of common mental disorders into the Primary Care Stroke Form, regular support and supervision from specialist teams to Primary Health Care, the development of liaison with traditional healers, access to transport for outreach work and health education to the community including schools, workplaces and linking to the media. There have been a number of continuing education courses for primary care staff, on average about 50 staff trained each year, initially funded by the MOH continuing education budget donated by Save the Children Fund, latterly by the WHO country office, and the WHOCC. The challenge remains to identify a sustainable budget for regular PHC continuing education, either within the national mental health programme or identified within DHMT budgets. Around 250 primary care staff have now received a one week CPD course, based on the WHO primary care guidelines, which have been adapted for Zanzibar, using consultation workshops and dialogue with the MOH, and are distributed during training, together with print outs and CDs of teaching slides, role plays, and the teacher's guide.

Considerable attempts have been made to obtain an adequate supply of essential medicines for primary care, with a number of meetings with the district health management teams (DHMTs) and the MOHSW. In 2004, the DHMTs identified 20 PHCs for a pilot trial of distribution of psychotropics (funded by DANIDA) including amitryptyline and anti-epileptics, with the PHC staff encouraged to assess, diagnose, treat and refer where appropriate. Staff in the 20 pilot PHCUs have been prioritised for the CPD course, and are visited monthly by a visiting team of psychiatric nurses from KC (subject to availability of funds for fuel). The visiting specialist team review people with severe mental illness who have been discharged from KC, see new referrals, and discuss complex cases with the primary care team. Since this system was initiated, inpatient numbers at KC have fallen steadily from 120 to less than 50 inpatients at any one time, and the average length of stay is now only a week or two rather than many months.

For the last six months of 2010, the medication supply has been good but remains fragile, paid for by intermittent funds from the Ministry of Health and Social Welfare and DANIDA. There is a long term agreement between the MOHSW in Zanzibar and Medical Store in Dar es Salaam, Tanzania mainland that the Zanzibar MOHSW has to procure medication from them. However, stocks of psychotropics are frequently not available in the Medical Stores, even when the MOHSW has the funds with which to buy them.

The policy recommendation to replace the single category with around 12 categories of mental disorders in the information collection system has not yet been implemented, making current routine data from primary care still useless for mental health planning purposes. The current version of the Primary Health Care Stroke form includes 3 categories, namely " Mental health diseases", Epilepsy and substance abuse. However, the stroke form for specialist care does now include 12 categories of mental disorder.

The recommendation for regular liaison between primary and specialist care to discuss criteria for referral, discharge letters, shared care procedures, need for medicines, information transfer, and any other co-ordination issues, training, development of good practice guidelines and consideration of appropriate resources is now starting to happen through the regular visits of psychiatric nurses from KC to the 20 pilot sites. Neighbouring PHCUs adjacent to the pilot sites also make use of the regular specialist visits, by referring clients for those visits, and plans are now being made to extend coverage of the liaison programme to the rest of Unguja and Pemba, although availability of fuel for transport will remain a major constraint.

Measures to strengthen and decentralise the specialist service included recommendation of structural repair of KC, provision of an 8 bedded inpatient unit on Pemba, together with an adequate supply of food and essential medicines; regular fumigation; intensive rehabilitation; continuing education and good practice guidelines; and a sustainable human resource strategy. Since the policy was adopted, there has been significant decentralisation, increased access to local care (initially at district outpatient clinics, but now directly at PHCU level in 20 pilot sites (12 on Unguja and 8 on Pemba), supported by training, good practice guidelines and some improvement of physical infrastructure. There has been specialist outreach support to the 20 pilot PHCUs (12 Unguja and 8 Pemba). Eight inpatient beds were allocated for acute admissions in Chake Chake hospital, Pemba, thus avoiding transfers to Unguja. Funding was allocated within the ADB lending programme for a purpose built unit in 2000 but has been subject to multiple delays and will be built this year at Wete, as a 20 bedded wing of the general hospital.

KC was renovated in 1999, and again deteriorated rapidly due to termites and flooding, but the female ward has now been successfully renovated again in 2008 with government and donor funds; and the ward toilets have been reengineered for easier maintenance. The water supply was improved with a bore hole in 2001 with donor funds, communication in the hospital wards was improved by installation of a landline funded by MOHSW, mosquito nets were obtained from a donor but are now regularly supplied by MOHSW, and the hospital is now regularly fumigated. The hospital van was repaired in 2003, which facilitated the transfer of patients to the main hospital and also general national activities by the mental health programme, but it broke down again in 2004 and was replaced by another former ambulance from Mnazi Moja in 2008.

The number of the patients participating in the Occupational Therapy Unit (OT) activities was increased and there was an increase in the ward-based activities. Only 10 patients per day attended OT in 1997, but has increased from time to time to around 30, depending on how many volunteers/students available to support the KC occupational therapist. Following the training of 3 nurses in the three year Occupational Therapy course at Moshi, on the Tanzania Mainland, occupational therapy services are now also available at Mnazi Moja hospital and UWZ, with whom there is good collaboration.

A regular programme of continuing education at KC was initiated to develop psychosocial skills in the ward staff but because there is still often only one qualified nurse and two orderlies on duty for around 50-60 patients, so the nurses have limited time to engage in direct therapy. Good practice guidelines for taking a history, care planning, and psychosocial treatments were introduced by the WHOCC through the Continuing Education programme for the hospital staff at KC which was started in 2000, coordinated for a few years by a UNV psychiatrist but lapsed after his departure, and has recently been reinvigorated. Textbooks were supplied to the KC library and to the College of Health Sciences by the WHOCC.

The quality of OT delivered has improved in its detailed assessment, planning and variety. Radio, TV, football and other games are also now available, as well as farming on the hospital land. The supply of medication at KCH, funded by DANIDA, for many years was sporadic and insufficient, because the Medical Store Department on the mainland fails to deliver on time, leading to prolonged inpatient stays, However, in the last 12 months, the medicine supply has been much better, and this combined with the outreach programme which delivers medication to the primary care clinics for clients living at home, has resulted in greatly reduced occupancy of KC (60 instead of 120 patients). The hospital food supply continues to fluctuate, vulnerable to rising food costs and the restricted funds received from Mnazi Moja. Physical care of inpatients was improved by the posting of a medical officer to KCW, resulting in reduced annual mortality of inpatients.

The policy proposed the establishment of a sustainable human resource and development strategy, but there has been no systematic resource for this. There are now 463 psychiatric nurses in Unguja and Pemba are 463, produced at a rate of 10-20 per year. A number of mental health nurses have received advanced post-basic training on the mainland, in the US and UK. Three nurses received the three year OT training at Moshi and two members of the mental health coordination team attended the advanced psychiatric nursing course at Dodoma. One nurse obtained a masters in the UK on substance abuse. One doctor is now being trained as a psychiatrist in Cuba. Meanwhile the semi-retired psychiatrist sees about 500 cases a year, or 10 a week, with severe mental illness.

The policy proposed strong coordination between the mental health programme and the substance abuse programme in order to tackle the co-morbidity between drug abuse and mental illness and this has happened, with close liaison with the NGO Zayadesa (Zanzibar youth against drugs, education and substance abuse ), and establishment of outreach and counselling services and a VCT clinic for drug users.

Measures to further strengthen community linkages included recommendations for dialogue with traditional health practitioners, and health education for the community. The MOHSW has met with THPs and the 20 pilot PHCs have been asked to initiate dialogue with THPs to encourage early referral of severe cases and discourage harmful practices. Some traditional healers have asked PHCUs for assistance with the further management of clients with malaria, psychosis and epilepsy. A vigorous health education programme for the community has been conducted over the last ten years, including talks in schools, workplaces, TV and radio, with district and national celebrations of World Mental Health Day, and mental health events linked to the Zanzibar Film Festival. Primary care workers have conducted local village visits, giving talks to schools and the general community, thus achieving good population coverage. Good cooperation has been established between the Health Education Unit, the Ministry of Education, the Department of Drug Control Programme and the Mental Health Programme. Direct collaboration has been established with the Youth and Child Welfare department in the ministry of women and children's affairs, and the director of the YCWD is now a member of the Mental Health Board.

Policy recommendations for learning disability services have resulted in liaison between the MOHSW, the Ministry of Education (MOE) and the learning disability NGO. The MOHSW and MOE have carried out joint trainings on the management of people with learning disabilities, training for teachers, and have developed a client needs assessment form. Activities remain limited by budgetary constraints, and need to be incorporated into routine budgets. It has not yet been possible to establish a dedicated clinic for the assessment and management of children with intellectual disability including a child psychologist and speech therapist, in collaboration with the dept of paediatrics at MM. However the MOE has now opened resource centres in almost all districts, to give support to teachers at all levels, from pre-school to secondary levels, about the care of children with intellectual disability. The mental health programme has taught the teachers in 5 resource teaching centres on Unguja and 4 on Pemba, each with 45 teaching staff, on normal child development, conduct disorders, emotional disorders and substance abuse. Training manuals have been produced and disseminated for teachers about mental health problems in children, which were funded by UNDP through the MOE. UNICEF (2008) sponsored a survey of intellectual disability in 4 districts, which assessed 1994 children and brought them into school under the Inclusive Education Programme. This inclusive education programme had been established in 1996 and gradually implemented thereafter as part of the Zanzibar Education master plan. The 'Zanzibar Poverty Reduction Plan: Basic Education and Skills Development'. Government of Zanzibar 2002 reported that although efforts have been made to promote equalization of opportunities in the field of education, disabled children are still a disadvantaged group in Zanzibar and still have limited access to education. Few schools cater for their specific needs and are mainly located in urban areas with only few qualified teachers to assist disabled children [23]. In addition, a subsequent case-finding survey of Zanzibar Town has identified a further 1,000 children with intellectual disability.

Policy recommendations on intersectoral liaison proposed national and local liaison between the prisons, police and MOHSW, and training for prison staff and police on mental health issues. The national mental health coordination team have established regular meetings with the Prison Commissioner, and have a Prison service department representative on the National Mental Health Board. The specialist staff at KC hospital are in communication with the Prisons department, and have established reliable methods of referral for prisoners who need regular follow up in the outpatient clinics or who need inpatient admission. Regular education and support for prison officers and prison nurses in recognition of mental disorders and criteria for referral to hospital has been achieved by dint of inviting the prison nurses to continuing education sessions for primary and specialist nurses, but the former prison commissioner would not allow the mental health staff to enter the prison. The mental health good practice guidelines have also been distributed to prison nurses. There is extensive liaison with police on the drug prevention programme, and some police nurses have attended primary care CPD courses, and been supplied with the good practice guidelines. For both prison staff and police, there is a major need for specific educational programmes about mental health issues.

The new policy explicitly encouraged liaison with relevant NGOs. The mental health NGO, SWAZA, has grown from strength to strength during the last ten years, with regular meetings, fundraising and activities, and is in active dialogue with the MOHSW. The Global Fund supported SWAZA to conduct home visits for mentally ill people, families and neighbours, to advise about mental health, substance abuse and HIV, and the risks of sexual abuse; about prevention of HIV and about how to care for people with mental illness and children with learning disabilities. SWAZA has also conducted a Football Bonanza for teenagers, with a football competition whereby the finalist teams compete in front of the House of Representatives to a large audience who are all given talks about substance abuse, and about how to be a good footballer without taking drugs or exposure to HIV risk. The message is promulgated that good football players are free from drugs and alcohol. SWAZA has also been given funds by UNODC to go to the 9 teacher training centres in Unguja and Pemba to teach the trainee teachers about mental health and substance abuse and how to recognise, and refer and advise.

A number of additional NGOs have now worked with the Mental Health Programme, including Zanzibar Information Against Drugs and Alcohol Abuse(ZIADA); ZASARNET (Zanzibar Substance Abuse Re-education Network-unfortunately now no longer operational); Youth Society in Zanzibar (TAQWA), especially on primary prevention of psychoactive abuse in youth, where they have worked together on the development of a training manual and training programme for peer trainers, and the design of brochures and banners for public information, and the delivery of health education about substance abuse through the media, community visits and schools; Zanzibar's Association of the Child 's Advocate (ZACA); and Zanzibar Association of Disability (UWZ). Training was delivered recently to the community based rehabilitation field officers, covering mental disorders, causes, early identification of children with intellectual disabilities, epilepsy, autism, referral to specialist services, and long term support to child and family. The KC specialist services and Community Based Rehabilitation field assistants carried out joint home visits to patients with complex needs living at home. Good communication has been achieved with the mental health association of Tanzania (MEHATA) and there has been mutual exchange and technical support, with collaboration on primary care guidelines and primary care training programmes. Zanzibar association of the parents of children with developmental disabilities (ZAPDD) is working with mental health programme and inclusive education-visiting schools to assess learning capacity of children and advise the parents and teachers on how to support their children in learning.

The mental health programme collaborates with the annual Zanzibar International Film Festival, working together since 1998 on community participatory approaches to mental health. Mental health talks have formed part of the annual Village Panorama. SWAZA has provided regular advocacy to the MOHSW, given extensive support to KCW by obtaining money from local donors for a bore hole to establish a water supply, hospital repairs, and a consultant led outpatient clinic is run by the retired psychiatrist who is chair of SWAZA. The mental health programme has a good relationship with Red Cross International of Zanzibar, collaborating on World Mental Health Day.

There has been extensive collaboration with the WHOCC in London which has given systematic technical support, occasional funds, regular dialogue, appraisal and review with the MOHSW, coordinators, staff of primary and specialist care, traditional healers, administrators and members of SWAZA; and also with the WHO liaison office in Zanzibar, which has supported the mental health programme through its biennual budget, and with the WHO country office on the mainland. VSO supplied two volunteers for KCH, and the United Nations Volunteer service (from UNDP) supplied a psychiatrist each for Unguja and Pemba for a few years (2001-4). Cuba has now deployed a psychiatrist to KC for two years, and is funding the training of a psychiatrist in Cuba. The presence of a psychiatrist in KC and on Pemba makes a significant difference to the quality of patient assessments and management, to patient survival, and to the training courses run at the College of Health Sciences.

The policy proposed the establishment of a computerised mental health information system covering needs, service inputs and processes, and health and social outcomes. In particular, the primary and secondary care information systems need to incorporate a more substantial mental health component so that they can serve as a basis for adequate planning and monitoring. Secondary care information has been improved but primary care information remains inadequate with all categories of mental disorder still recorded in a single category, despite earlier agreement to include a more detailed breakdown. A national mental health report has been produced annually since 2003, and its content expanded. Suicide data is now collected by the police, but there is still a need to include the cause of death, diagnoses and information from health records. The law criminalizing suicide attempts still needs review. The policy proposed that deaths from physical illness in the inpatient units should be carefully monitored and audited, and this has improved to some extent but more could be done. The policy proposed systematic public education on mental health and mental disorders, and cooperation has been established between the Health Education Unit, the Ministry of Education, the Department of Drug Control Programme and the Mental Health Programme. Health education has been delivered via visits to villages to carry out talks to schools and the general community, radio programmes and TV programmes. The village visits were conducted by primary care workers, thus achieving good coverage of the islands.

The policy proposed the development of a suicide prevention strategy. An audit of all suicides is needed to gain a better understanding of causes and means of suicide. The mental health programme has held discussions with the Department of Information and Media to encourage more responsible reporting of suicide, but this remains problematic. Education of primary and secondary care teams about assessment and management of suicidal risk and support to high-risk groups has been included in the CE programmes, and is now being included in the college curriculum for basic training. More needs to be done to support high risk groups, and to change the legislation on attempted suicide to make it easier to access help.

## Discussion

The request of the MOHSW to WHO for technical assistance in 1997 provided an entry point for the WHOCC to provide long term technical support to Zanzibar to move from a mental health programme entirely focussed on the national mental hospital to a more decentralised holistic vision for the mental health of Zanzibar. The early agreement of the MOHSW to establish the mental health coordination team (see additional file 3) has been key to systematic governance and implementation of the programme. The establishment and growth of SWAZA has also been crucial in providing local support for the implementation of the programme, including mobilisation of local donor funds, and continued advocacy to the MOHSW.

Implementation of the policy has been very challenging; it has needed iterative and sustained discussions with the MOHSW to develop the holistic vision for the evolution of services, to perceive that change was feasible, and that multiple interventions were possible and realistic. Resource constraints have been severe across the whole of the health and social sectors, but especially for mental health which is not a priority for any major international donor [24]. As well as lack of financial resource, lack of human resource is critical. As in other countries, Zanzibar struggles to expand its health personnel. It trains nurses locally, and this has provided the backbone of the health service. Its decision in the 1980s to give all student nurses a 4^th ^year of either psychiatry or midwifery was inspired and has led to the production of able staff who are able to deliver mental health services. It will need to be seen whether the recent reduction to 3 years will reduce the psychiatric competence of the staff produced.

The government of Zanzibar has to send students to other countries to train as doctors, and to specialise, including as psychiatrists. Such overseas training risks brain drain [25], and the length of time taken to produce a psychiatrist makes it a heavy investment (around 6 years of medical training followed by 4 years of psychiatry) for a small country to make, only to risk loss to a wealthier country at the end of the training. Nonetheless Zanzibar is now training another psychiatrist to fill the gap left by the first who retired in the mid 90s. The MOHSW also needs to urgently initiate training of a second psychiatrist for Pemba. There is no doubt that a psychiatric presence can greatly influence the quality of assessments and interventions by the nurses, and is always desirable. While UNV supplied a psychiatrist each to Unguja and Pemba in the early 2000s this provision could only ever be short term, as UNV rules prevent its extension, so it is essential for Zanzibar to become self sufficient in terms of psychiatric leadership.

Sustainability of human resource is a national problem not only for the health service. Many reasons contribute to health staff leaving Zanzibar, but the main one is low pay, even relative to Tanzania mainland. A junior Nurse with a diploma in the Mainland earns a basic salary of about 600,000/-T Shillings compared to only 150,000/-T Shillings in Zanzibar. In addition, there are no incentives to encourage staff in Zanzibar to choose the mental health field rather than other speciality areas or primary care, and work overload in mental health care is considerable.

Much still remains to be done. Funding for CPD and for supervision to PHCUs remain the priorities, and for this access to transport by the senior specialist staff is essential. Despite enormous efforts it has not been possible to improve the food supply from MM to KC for the patients whose diet remains rice and beans; however the long term impact of such dietary restriction is now greatly mitigated by the fact that inpatient stay is now usually a few weeks rather than many months or years. Nonetheless it remains imperative that KC should have its own budget, separated from that of MM, otherwise it will always find its annual allocation drained by MM, with an obvious lack of equity between the psychiatric and the medical and surgical inpatients. Nonetheless our long term follow up suggests that despite extremely difficult resource constraints it has proved possible to make significant progress, with decentralised services, integration into primary care, and community education. Further progress would be greatly enhanced by better inclusion of mental health into mainstream health sector funding and developments such as HMIS. It has proved difficult to get mental health properly integrated into HMIS in other East African countries as well, encountering strong resistance from health sector reform teams which tend to be dominated by communicable disease specialists [25].

It is likely that once such integration is achieved, considerable progress will follow, as the relative priority of mental disorders will be clear in the annual routine health service dataset [26]. This year, mental health is clearly specified in the Zanzibar national health sector strategy and this may lead to better funding flows.

## Conclusions

A multi-faceted and comprehensive programme can be effective in achieving considerable strengthening of mental health programmes and services even in extremely low resource settings, but requires sustained input and advocacy if gains are to be maintained and enhanced.

## Competing interests

The authors declare that they have no competing interests.

## Authors' contributions

RJ led the first draft and, MM, SH, MH, AS, SS, AR, AW, JM contributed to subsequent drafts. All authors read and approved the final manuscript.

## Supplementary Material

Additional file 1 **The Implementation Process**. Click here for file

Additional file 2 **Outputs and activity components of the Zanzibar mental health programme**. Click here for file

Additional file 3 **Organogran for Implementation of the National Mental Health Plan**. Click here for file
