# Supplementary material for: Developing and implementing mental health policy in Zanzibar, a low income country off the coast of East Africa
Source: Int J Ment Health Syst. 2011 Feb 14;5:6. doi: 10.1186/1752-4458-5-6 (PMC3045977; doi:10.1186/1752-4458-5-6)
Supplement: Additional file 1 — The Implementation Process. [file 1752-4458-5-6-S1.DOC]

| **Additional file 1 -** **The Government is seeking to achieve these goals by five major domains of work:** | |
| --- | --- |
| 1. | Giving the implementation of the mental health policy the status of a special programme, with direct accountability to the Deputy Principal Secretary and the Minister of Health |
| 2. | Strengthening and developing existing systems for education, human resource development, information and communication |
| 3. | Developing the existing primary care system |
| 4. | Transforming the existing secondary care system into a more flexible approach for Zanzibar’s needs |
| 5. | Linking to existing educational programmes in schools, workplaces, the community and the media |
