# Supplementary material for: Developing and implementing mental health policy in Zanzibar, a low income country off the coast of East Africa
Source: Int J Ment Health Syst. 2011 Feb 14;5:6. doi: 10.1186/1752-4458-5-6 (PMC3045977; doi:10.1186/1752-4458-5-6)
Supplement: Additional file 2 — Outputs and activity components of the Zanzibar mental health programme. [file 1752-4458-5-6-S2.DOC]

**Additional file 2 - Outputs and activity components of the Zanzibar mental health programme**

| **Output 1. To enhance the capability of the ministry to implement the mental health programme** | | |
| --- | --- | --- |
| **Activities** | |  |
| **1.1** | | The appointment of a national mental health coordinator |
| **1.2** | | The establishment of the national mental health implementation committee |
| **1.3** | | The establishment of monthly coordination meetings between the national coordinator, the 2 island coordinators and the3 sector coordinators for each island |
| **Output 2. To strengthen primary mental health care services** | | |
| **Activities** |  | |
| **2.1** | The inclusion of mental health into the continuing education programmes for primary care staff | |
| **2.2** | The development of good practice guidelines | |
| **2.3** | An adequate supply of essential medicines | |
| **2.4** | The inclusion of common mental disorders into the primary care stroke form | |
| **2.5** | Regular liaison with specialist teams | |
| **2.6** | The development of liaison with traditional healers | |
| **2.7** | Access to transport for outreach work | |
| **2.8** | Health education to the community including schools, workplaces and linking to the media | |
| Output 3. To rationalise and strengthen the secondary mental health care system | | |
| **Activities** |  | |
| **3.1** | Modernise the existing inpatient unit on Unguja (Kidongochekundu Hospital) | |
| **3.2** | Establish an inpatient unit on Pemba | |
| **3.3** | Link both inpatient units to their respective outpatient centres and give liaison support to local primary health care units | |
| **3.4** | Provide necessary support services/materials. Including ECT and EEG machines, and OT materials and fuel allowance for outings for patients | |
| **3.5** | Develop good practice guidelines for use in inpatient units and outpatient clinics | |
| **3.6** | Ensure adequate supply of essential medicines | |
| **3.7** | Ensure adequate supply of food to inpatient units | |
| **3.8** | Ensure adequate supply of fumigation chemicals | |
| Output 4. to establish effective links between primary and specialist care | | |
| **Activities** |  | |
| **4.1** | The primary care system needs regular liaison with specialist care to discuss criteria for referral, discharge letters shared care procedures, need for medicines, information transfer, and any other co-ordination issues, training, development of good practice guidelines and consideration of appropriate resources | |
| Output 5. Ensure an adequate supply of essential medicines for PHCUs, OPDs and inpatient unit | | |
| **Activities** |  | |
| **5.1** | Deliver an adequate supply of essential medicines to PHCUs, OPDs and inpatient units | |
| **Output 6. Develop good practice guidelines for PHCUs, OPDs and inpatient units** | | |
| **Activities** |  | |
| **6.1** | Good practice guidelines are an invaluable adjunct to improving care and establishment of good practice at all times | |
| Output 7. To serve the mental health needs of women and children | | |
| **Activities** |  | |
| **7.1** | Develop accessible and effective services for women and children because women and children are a precious resource for the nation | |
| **7.2** | In addition to the general adult rates of illness, women also experience higher rates of illness around the time of childbirth. If untreated, these disorders can affect the mother’s relationship with her children, thus damaging the child’s cognitive and emotional development | |
| Output 8. Develop services for people with intellectual disability | | |
| **Activities** |  | |
| **8.1** | Explicit liaison between the Ministry of Health and the Ministry of Education | |
| **8.2** | Establish a dedicated clinic for the assessment and management of children with intellectual disability including a child psychologist and speech therapist | |
| **8.3** | Establish an Education Resource Centre | |
| **8.4** | Develop a teaching module for inclusion in the basic training and continuing primary care education | |
| **8.5** | Good practice guidelines for primary care and for teachers | |
| **8.6** | Liaison with the NGO, SWAZA, and with the NGO, Watemavu | |
| Output 9. Establish a formal mechanism for close liaison between the mental health services and other governmental, non-governmental and traditional sectors. | | |
| **With Prisons and Police** | | |
| **Activities** |  | |
| **9.1 a** | There should be liaison, leadership and agreement at a senior level between the prison and the ministry of health | |
| **9.2 a** | Liaison between the mental health services and the prison | |
| **9.3 a** | Regular education and support for prison officers and prison nurses about the recognition of mental disorders and criteria for referral to hospital | |
| **9.4 a** | Liaison, leadership and agreement at a service level between the police and the ministry of health | |
| **9.5 a** | Liaison between primary health care units, outpatient clinics and local police | |
| **9.6 a** | Education for police officers about mental health, procedures for transfer to hospital | |

| **With NGO’s** | |
| --- | --- |
| **Activities** |  |
| **9.1 b** | People with mental illness, their carers and the community are the “Customers” of the services. Their involvement can greatly improve the planning and delivery of services as they can spot gaps and problems as well as comment on what is working well. Pump priming the NGO can be a cost-effective way of achieving progress |
| **With Traditional Healers** | |
| **Activities** |  |
| **9.1 c** | Liaison between national mental health coordinator, island coordinators, sector coordinators and the leaders of the traditional healer professional association |
| **9.2 c** | Traditional healers already see many people with mental disorders and it will be helpful to create regular opportunities for mutual discussion |
| **9.3 c** | Agreement of collaborative ways of working which may eventually include criteria for referral, use of diagnostic algorithms, shared care and consideration of appropriate research |
| **Output 10. Promote the mental health of Zanzibari people** | |
| **Activities** |  |
| **10.1** | Existing health education programmes in schools, workplaces and the community need to incorporate substantial mental health component |
| **10.2** | To enhance the understanding of mental health within the ministry of Information, Tourism and Youth and an appreciation of the important contribution which this ministry can make to the overall mental health of Zanzibar in relation to the role of the media, visits of foreigners, and activities of young people who are the greatest manpower of the country |
| **10.3** | To coordinate the mental health programme and the substance abuse programme to tackle the co-morbidity between drug abuse and mental illness |
| **10.4** | Enhance effectiveness in community education and prevention programmes |
| Output 11. Establish a computerised mental health information system covering needs, service inputs and processes, and health and social outcomes | |
| **Activities** |  |
| **11.1** | The primary and secondary care information systems need to incorporate a more substantial mental health component so that they can serve as a basis for adequate planning and monitoring |
| **11.2** | This includes addition of the main broad categories of disorder to the primary care stroke form |
| **11.3** | A mental health report should be produced annually. Deaths from suicide should be officially collected |
| **11.4** | Deaths from physical illness in the inpatient units should be carefully monitored |

| Output 12. Establish a research and development strategy | |
| --- | --- |
| **Activities** |  |
| **12.1** | It is necessary to facilitate both access to research from other countries by access to journals and the use of the internet and to strengthen the existing local research programme |
| Output 13. Establish a sustainable human resource and development strategy | |
| **Activities** |  |
| **13.** | Zanzibar, as a relatively small country, cannot meet all its training requirements for health and social care professionals. It therefore needs a sustainable plan for production and continuing development both at home, on the mainland and elsewhere, of primary and secondary care staff. |
| Output 14. Develop strategy to reduce suicide | |
| **Activities** |  |
| **14.** | Suicides are an avoidable mortality and appropriate action includes audit, work with the media, reduction of access to means, education of primary and secondary care teams about assessment and management of suicidal risk and support to the high risk groups |
| Output 15. Strategy to reduce physical mortality in people with mental illness | |
| **Activities** |  |
| **15.1** | The mortality in people with severe mental illness in Zanzibar is very high, and action is needed to improve the physical condition of the inpatient unit, including soap and water and drains, hygienic procedures and cooking facilities |
| **15.2** | to improve the physical health assessments and care |
| **15.3** | Prompt action in cases of suspected cholera |
| Output 16. A new mental health act is needed to support the implementation of Zanzibar's mental health policy | |
| **Activities** |  |
| **16.1** | Pass legislation |
| **16.2** | Prepare practical guidance for staff, information for patients ,relatives and the community including police |
