# Supplementary material for: Developing and implementing mental health policy in Zanzibar, a low income country off the coast of East Africa
Source: Int J Ment Health Syst. 2011 Feb 14;5:6. doi: 10.1186/1752-4458-5-6 (PMC3045977; doi:10.1186/1752-4458-5-6)
Supplement: Additional file 3 — Organogran for Implementation of the National Mental Health Plan. [file 1752-4458-5-6-S3.DOC]

**Additional file 3 - Outputs and activity components of the Zanzibar mental health programme**

**Minister**

**Deputy Principal Secretary**

**National mental health co-ordinator**

**And**

**(Administrative and manpower development officer)**

# Unguja mental health co-ordinator Pemba mental health co-ordinator

(Psychiatrist) (Psychiatrist)

| **Specialist Sector Coordinator** | **Primary Care Coordinator** | **Community Coordinator** | **Specialist Sector Coordinator** | **Primary Care Coordinator** | **Community Coordinator** |
| --- | --- | --- | --- | --- | --- |
| Inpatient Beds | PHCUs | NGOs | Inpatient beds | PHCUs | NGOs |
| Occupational rehabilitation | Liaison with traditional healers | Schools | Occupational rehabilitation | Liaison with traditional healers | Schools |
| OPDs |  | Workplaces | OPDs |  | Workplaces |
| Liaison with police and prisons |  | Liaison with health education | Liaison with police and prisons |  | Liaison with health education |
| Continuing training | Continuing training | Liaison with addiction programme | Continuing training | Continuing training | Liaison with addiction programme |
